# Fast Second Degree Total Variation Method for Image Compressive Sensing

**DOI:** 10.1371/journal.pone.0137115

**Published:** 2015-09-11

**Authors:** Pengfei Liu, Liang Xiao, Jun Zhang

**Affiliations:** 1 School of Computer Science and Engineering, Nanjing University of Science and Technology, Nanjing, Jiangsu, China; 2 School of Science, Nanjing University of Science and Technology, Nanjing, Jiangsu, China; Beijing University of Technology, CHINA

## Abstract

This paper presents a computationally efficient algorithm for image compressive sensing reconstruction using a second degree total variation (HDTV2) regularization. Firstly, a preferably equivalent formulation of the HDTV2 functional is derived, which can be formulated as a weighted *L*_1_-*L*_2_ mixed norm of second degree image derivatives under the spectral decomposition framework. Secondly, using the equivalent formulation of HDTV2, we introduce an efficient forward-backward splitting (FBS) scheme to solve the HDTV2-based image reconstruction model. Furthermore, from the averaged non-expansive operator point of view, we make a detailed analysis on the convergence of the proposed FBS algorithm. Experiments on medical images demonstrate that the proposed method outperforms several fast algorithms of the TV and HDTV2 reconstruction models in terms of peak signal to noise ratio (PSNR), structural similarity index (SSIM) and convergence speed.

## Introduction

Recently, the compressive sensing (CS) technique which condenses the information in sparse or compressible images into a small amount of data and yet reconstructs them accurately has been developed for data acquisition in many practical applications, including radar receivers, magnetic resonance imaging (MRI) and microscopy [1,2].

In this paper, we focus on the issue of reconstructing a desired image *f*:Ω→ℜ from its noisy and undersampled Fourier measurement *g*, where Ω⊂ℜ^2^ is the spatial support of the image. In general, the observed measurement *g* is often linearly modeled as

g=Af+e, (1)

where *A* is a linear operator representing the randomized partial Fourier sampling pattern, and *e* is often assumed to be Gaussian white noise with standard deviation σ. As is well known, the reconstruction of *f* from its observed measurement *g* is an ill-posed problem. Variational regularization approach is usually employed to cope with the ill-posedness by formulating image CS reconstruction as an optimization problem:

$$\hat{f}=\underset{f}{\text{arg min}}\left\{C(f)=\frac{1}{2}{\int_{\Omega }\left|g-Af\right|}^{2}dxdy+\lambda \;J(f)\right\},$$ (2)

where the first term is called the data fidelity term, while the second one *J*(*f*) is called the regularization term which enforces some prior knowledge on the desired image, and the regularization parameter *λ*≥0 balances the contributions of the two terms.

As an important and valid sparsity regularizer, the total variation (TV) regularizer defined by the *L*_1_ norm of image gradient has been applied to image CS reconstruction problems and turned out to be very efficient for preserving image edges [3–5]. However, TV favors piecewise constant solutions so that TV-based CS reconstruction methods easily tend to produce severe staircase effect in the smooth regions of reconstructed image. To reduce the staircase effect and preserve image edges sharpness as far as possible, higher order regularization schemes have been gradually investigated by more and more researchers for linear inverse problems [6–11]. Recently, a novel family of higher order regularizers termed as higher degree total variation (HDTV) has been proposed for image CS reconstruction, and their experimental results demonstrate that HDTV can effectively minimize the staircase effect while better preserving the edges and singularities [12]. Specifically, HDTV preserves strong directional derivatives at a specified direction but attenuates the small ones at other directions thus encouraging smoothing along line-like features and enhancing them. Moreover, the use of higher degree derivatives yields piecewise linear solutions so that they can minimize the staircase effect and provide state-of-the-art results.

In spite of this, the main challenge of the current HDTV method consists in its high computational complexity compared to the popular TV methods. Due to the non-differentiability of HDTV, the corresponding HDTV-based reconstruction model proposed in [12] is solved by using a majorization-minimization (MM) algorithm, which is similar to the iteratively reweighted algorithms used in the TV minimization problems [13]. Under the MM framework, the MM algorithm proceeds by successively minimizing a sequence of quadratic surrogate functionals that majorize the HDTV regularizer and the data fidelity term, then the minimization of quadratic surrogate functionals is solved by using a conjugate gradient (CG) algorithm. Specifically, the iteratively reweighted MM algorithm alternates between the CG optimization and the update of the weights from current iterate. However, the spatially varying weights often tend to be very large values, then the minimization subproblems of quadratic surrogate functionals are with large condition numbers. As a result, the iteratively reweighted MM algorithm is computational expensive so as to result in slow convergence speed. To reduce the computational complexity and accelerate the convergence speed, we are motivated to investigate more efficient HDTV-based algorithms.

In this paper, we only consider the special case of second degree total variation (HDTV2)-based reconstruction model and focus on introducing a computationally efficient HDTV2 reconstruction algorithm. Compared to the previous work [12], our main contributions are listed as follows:

1. Under the spectral decomposition framework, we reinterpret the original HDTV2 regularizer which is the *L*_1_-*L*_2_ mixed norm of the second degree directional derivatives as a novel weighted *L*_1_-*L*_2_ mixed norm of the second degree image derivatives.
2. To facilitate computation, we design an efficient forward-backward splitting (FBS) scheme to solve the equivalent minimization formulation of HDTV2 reconstruction model under additional convex constraints.
3. A complete convergence proof of the proposed FBS algorithm is shown relying on the properties of averaged and firmly non-expansive operators. The experimental results of our proposed scheme compared with the recently iteratively reweighted MM scheme [12] demonstrate the significant performance improvements in terms of peak signal to noise ratio (PSNR), structural similarity index (SSIM) and convergence speed.

The rest of this paper is outlined as follows. In next section, we first review the HDTV2-based image reconstruction model and analyze in detail the shortcomings of the iteratively reweighted MM scheme for HDTV2-based reconstruction model. To overcome these shortcomings, a novel equivalent formulation of HDTV2 is derived. Owing to the equivalent formulation, an efficient algorithm using FBS scheme is introduced for HDTV2-based minimization problem and the convergence of the proposed FBS algorithm is also proved. Section Experiments shows the experimental results for medical image CS reconstruction problems. Finally, we conclude the paper in Section Conclusions.

## Second Degree Total Variation (HDTV2)-Based Image Reconstruction Model

In this paper, we just consider the special case of HDTV corresponding to the second degree total variation (HDTV2) for image reconstruction.

### Review and analysis of HDTV2-based reconstruction model

As detailed in [12], the HDTV2-based reconstruction model is specified as

$$\hat{f}=\underset{f}{\text{arg min}}\left\{R(f)=\frac{1}{2}{\int_{\Omega }\left|g-Af\right|}^{2}dxdy+\lambda \;HDTV_{2}(f)\right\},$$ (3)

where *HDTV*_2_(*f*) is the HDTV2 regularizer defined as the *L*_1_-*L*_2_ mixed norm of second degree directional derivatives of image *f*, which is formulated as

$$HDTV_{2}(f)=\int_{\Omega }||f_{\theta ,2}(x,y)|{|}_{L_{2}[0,2\pi ]}dxdy=\int_{\Omega }\sqrt{\frac{1}{2\pi }\int_{0}^{2\pi }{\left|f_{\theta ,2}(x,y)\right|}^{2}d\theta }\;dxdy,$$ (4)

with

$$f_{\theta ,2}(x,y)=\underset{S_{2}^{Τ}(\theta )}{\underset{︸}{\left[{\text{cos}}^{2}\theta ,2\text{cos}\theta \text{sin}\theta ,{\text{sin}}^{2}\theta \right]}}\;\underset{G_{2}(x,y)}{\underset{︸}{\left[\begin{array}{l} \;\partial^{2}f(x,y)/\partial x^{2}\\ \partial^{2}f(x,y)/\partial x\partial y\\ \;\partial^{2}f(x,y)/\partial y^{2} \end{array}\right]}},$$ (5)

denoted the second degree directional derivative of image *f* at coordinate (*x*, *y*) along the unit vector **u**_*θ*_ = [cos*θ*,sin*θ*]^T^, where **G**_2_(*x*, *y*) is a vector consisting of the second degree partial derivatives of image *f* at coordinate (*x*, *y*), and (·)^T^ denotes the transpose operator.

Based on the definitions (4) and (5), the HDTV2 regularizer can be rewritten as

$$HDTV_{2}(f)=\int_{\Omega }\sqrt{G_{2}^{Τ}(x,y)C_{2}G_{2}(x,y)}dxdy,$$ (6)

where $C_{2}=\frac{1}{2\pi }\int_{0}^{2\pi }S_{2}(\theta )S_{2}^{Τ}(\theta )d\theta \in {ℜ}^{3\times 3}$ is a symmetric matrix with entries $C_{2}(i,j)=\frac{1}{2\pi }\int_{0}^{2\pi }S_{2}^{\left(i\right)}(\theta )S_{2}^{\left(j\right)}(\theta )d\theta$ , *i*, *j* = 1,⋯,3, and $S_{2}^{\left(i\right)}(\theta )$ denotes the *i* ^*th*^ element of **S**_2_(*θ*) such that $C_{2}=\frac{1}{8}\left[\begin{array}{l} \;3\;0\;1\\ 0\;4\;0\\ \;1\;0\;3 \end{array}\right]$ .

Since HDTV2 is nondifferentiable, the authors in [12] use an iteratively reweighted MM algorithm to solve model (3) at *k* ^*th*^ iterate as follows:

$$\left\{ \begin{array}{l} f^{\left(k+1\right)}=\underset{f}{\text{arg min}}\left\{\frac{1}{2}{\int_{\Omega }\left|g-A\;f\right|}^{2}dxdy+\lambda \;\int_{\Omega }G_{2}^{Τ}(x,y)E^{\left(k\right)}(x,y)G_{2}(x,y)dxdy\right\}\\ E^{\left(k\right)}(x,y)=\frac{1}{2\sqrt{G_{2}^{\left(k\right)}{}^{{}^{Τ}}(x,y)C_{2}G_{2}^{\left(k\right)}(x,y)}}C_{2} \end{array}\right.,$$ (7)

which then can be solved by using a conjugate gradient (CG) algorithm. Specifically, the MM algorithm alternates between CG optimization (computing *f* ^(*k*+1)^ subproblem) and the recomputation of the weights from the current iterate (computing **E** ^(*k*)^). However, the spatially varying weights in **E** ^(*k*)^ often tend to be very large values so that the *f* ^(*k*+1)^ subproblem is with large condition number. Hence, the resulting MM algorithm is also computationally expensive so as to show slow convergence speed.

To reduce the computational cost and accelerate the convergence speed, we introduce a more efficient HDTV2-based reconstruction algorithm. To this end, we first derive an equivalent formulation of HDTV2, and then use the equivalent formulation to design a computationally efficient HDTV2 reconstruction algorithm in the following sections.

### Reinterpretation of HDTV2

From now on, we will reinterpret the HDTV2 regularizer defined in Eq (4) as a weighted *L*_1_-*L*_2_ mixed norm of second degree image derivatives under the spectral decomposition framework.

As shown in Eq (6), since **C**_2_ is symmetric and positive definite, we can use the spectral decomposition theorem to rewrite **C**_2_ as

$$C_{2}\text{=}\underset{Q_{2}}{\underset{︸}{\left(\frac{\text{1}}{\sqrt{\text{2}}}\left[\begin{array}{ccc} \text{1} & \text{1} & \text{0}\\ \text{0} & \text{0} & \sqrt{\text{2}}\\ \text{-1} & \text{1} & \text{0} \end{array}\right]\right)}}\underset{D_{2}}{\underset{︸}{\left(\frac{\text{1}}{\text{4}}\left[\begin{array}{ccc} \text{1} & \text{0} & \text{0}\\ \text{0} & \text{2} & \text{0}\\ \text{0} & \text{0} & \text{2} \end{array}\right]\right)}}{\underset{Q_{2}^{Τ}}{\underset{︸}{\left(\frac{\text{1}}{\sqrt{\text{2}}}\left[\begin{array}{ccc} \text{1} & \text{1} & \text{0}\\ \text{0} & \text{0} & \sqrt{\text{2}}\\ \text{-1} & \text{1} & \text{0} \end{array}\right]\right)}}}^{\text{Τ}}$$ (8)

and we further obtain that $Q_{2}D_{2}^{1/2}Q_{2}^{Τ}=\frac{1}{4}\left[\begin{array}{ccc} \sqrt{2}+1 & 0 & \sqrt{2}-1\\ 0 & 2\sqrt{2} & 0\\ \sqrt{2}-1 & 0 & \sqrt{2}+1 \end{array}\right]$ which is also symmetric and positive definite.

Based on that, we will derive an equivalent formulation of the HDTV2 regularizer in the following proposition.

#### Proposition 1

Let $W_{2}=Q_{2}D_{2}^{1/2}Q_{2}^{Τ}=\frac{1}{4}\left[\begin{array}{ccc} \sqrt{2}+1 & 0 & \sqrt{2}-1\\ 0 & 2\sqrt{2} & 0\\ \sqrt{2}-1 & 0 & \sqrt{2}+1 \end{array}\right]$ and the second degree vectorial differential operator *∂*_2_ = (*∂*_*xx*_, *∂*_*xy*_, *∂*_*yy*_)^T^, then the HDTV2 can be formulated as

$$HDTV_{2}(f)=\int_{\Omega }||W_{2}\partial_{2}f(x,y)|{|}_{2}dxdy,$$ (9)

where || ⋅ ||_2_ stands for the Euclidean norm.

**Proof**: Combining Eqs (6) and (8), then HDTV2 can be simplified as

$$\begin{array}{l} HDTV_{2}(f)=\int_{\Omega }\sqrt{G_{2}^{Τ}(x,y)C_{2}G_{2}(x,y)}dxdy\\ \;=\int_{\Omega }\sqrt{G_{2}^{Τ}(x,y)Q_{2}D_{2}Q_{2}^{Τ}G_{2}(x,y)}dxdy\\ \;=\int_{\Omega }\sqrt{G_{2}^{Τ}(x,y)Q_{2}D_{2}^{1/2}Q_{2}^{Τ}\underset{W_{2}}{\underset{︸}{Q_{2}D^{1/2}Q_{2}^{Τ}}}G_{2}(x,y)}dxdy\\ \;=\int_{\Omega }||W_{2}\underset{\partial_{2}f(x,y)}{\underset{︸}{G_{2}(x,y)}}|{|}_{2}dxdy. \end{array}$$ (10)

As described in Eq (10), the HDTV2 regularizer can be reinterpreted as weighted *L*_1_-*L*_2_ mixed norm of second degree image derivatives, while **W**_2_ can be understood as the weighting matrix. According to the equivalent formulation of HDTV2, it is more preferable for the algorithm we will propose in next section.

## Forward-Backward (FB) Splitting Algorithm for HDTV2-Based Image Reconstruction

In this section, we focus on using the equivalent formulation of HDTV2 to design a more efficient HDTV2 reconstruction algorithm.

### Discrete Model Formulation

First of all, we will give the discrete formulation of model (3). To simplify our analysis, an image with size *r*×*c* is stacked in a vector of size *N* = *r*×*C*, we assume the reflexive boundary condition for images and use the forward finite differences to approximate the second degree derivatives [14].

Next, we define operator **V**_2_:ℜ^*N*^→ℜ^3×N^ to refer to the discrete version of the vectorial differential operator *∂*_2_ defined in proposition 1, and its adjoint operator is defined as $V_{2}^{Τ}:{ℜ}^{3\times N}\to {ℜ}^{N}$ . Hereafter, we denote *X*_2_ = ℜ^3×N^. Then, let **p** = (**p**_1_,**p**_2_,⋯,**p**_*N*_)∈*X*_2_ and **q** = (**q**_1_,**q**_2_,⋯,**q**_*N*_)∈*X*_2_, we define the inner product ${\langle \;\cdot \;,\;\cdot \;\rangle }_{X_{2}}$ and norm $||\cdot |{|}_{X_{2}}$ in *X*_2_ as ${\langle \;p,q\rangle }_{X_{2}}=\sum_{i=1}^{N}p_{i}^{Τ}q_{i}$ and $||q|{|}_{X_{2}}=\sqrt{{\langle \;q,q\rangle }_{X_{2}}}$ .

Moreover, let us define operator **Λ**_2_:*X*_2_→*X*_2_ and its adjoint operator $\Lambda_{2}^{Τ}:X_{2}\to X_{2}$ , that act on vector field **p** = (**p**_1_,**p**_2_,⋯,**p**_*N*_)∈*X*_2_ as (**Λ**_2_ **p**)_*i*_ = **W**_2_ **p**_*i*_ and ${\left(\Lambda_{2}^{Τ}p\right)}_{i}=W_{2}^{Τ}p_{i}$ , where (⋅)_*i*_ denotes the *i* ^*th*^ column element of the corresponding vector filed.

With the *l*_1_−*l*_2_ mixed norm of a vector field **p** = (**p**_1_,**p**_2_,⋯,**p**_*N*_)∈*X*_2_ defined as $||p|{|}_{1,2}=\sum_{i=1}^{N}||p_{i}|{|}_{2}$ , then the HDTV2 described in Eq (10) can be discretized as

$$HDTV_{2}(f)=\sum_{i=1}^{N}||W_{2}{\left(V_{2}f\right)}_{i}|{|}_{2}=\;||\Lambda_{2}V_{2}f|{|}_{1,2}.$$ (11)

In this paper, we concentrate on the issue of image reconstruction from undersampled Fourier data. Therefore, we can write the discrete formulation of the model (3) as

$$\hat{f}=\underset{f}{\text{arg min}}\left\{R(f)=\frac{1}{2}||g-Af|{|}_{2}^{2}+\lambda ||\Lambda_{2}V_{2}f|{|}_{1,2}\right\},$$ (12)

where **A** = **PF**∈*C* ^*M×N*^ is the sensing matrix with **F**∈*C* ^*N×N*^ denoted the two-dimensional discrete Fourier transform (DFT) matrix and **P**∈*C* ^*M×N*^ denoted the selection matrix containing *M* rows of the identity matrix of order *N*, **g**∈*C* ^*M*^ is the observed undersampled Fourier measurement and **f**∈ℜ^*N*^ is the image to be reconstructed.

In the following, we will describe in detail the minimization algorithm for solving above model (12).

### The Proposed Algorithm

Let us denote $R_{1}(f)=\frac{1}{2}||g-Af|{|}_{2}^{2}$ and *R*_2_(**f**) = *λ*||**Λ**_2_ **V**_2_ **f**||_1,2_, it is clear that *R*_1_ and *R*_2_ are both proper and lower semicontinuous convex functions from ℜ^*N*^ to [0,+∞), then the model (12) becomes the minimization of functions of the form *R* = *R*_1_+*R*_1_. Specifically, let *G* = {**f**∈ℜ^*N*^ |0∈∂*R*(**f**)} be the set of solutions of model (12), where ∂*R* is the subdifferential of function *R*, then **f**∈*G* if and only if

$$\begin{array}{l} 0\in \partial R(f)\Leftrightarrow 0\in \partial (\beta \;R)(f)\;\forall \beta >0\\ \;\Leftrightarrow f-f\in \partial (\beta \;R)(f),\\ \;\Leftrightarrow f=prox_{\beta \;R}(f) \end{array}$$ (13)

where *prox*_*βR*_:ℜ^*N*^→ℜ^*N*^ is proximity operator of function *βR*, which is defined as

$$prox_{\beta \;R}(f)=\underset{y}{\text{arg min}}\frac{1}{2}||f-y|{|}_{2}^{2}+\beta \;R(y),$$ (14)

where *β* is known as the proximal step size.

Thus, the proximal-type algorithm for model (12) can be recursively constructed as

$$f^{\left(k+1\right)}=prox_{\beta \;R}(f^{\left(k\right)}).$$ (15)

However, the main difficulty with above proximal-type algorithm is that *prox*_*βR*_ is hard to compute. Under the operator splitting framework, splitting methods for model (12) do not attempt to compute the proximal operator *prox*_*βR*_ of the combined function *βR*, but instead perform a sequence of calculations involving separately the individual proximal operators $prox_{\beta \;R_{1}}$ and $prox_{\beta \;R_{2}}$ which are relatively easier to compute.

As detailed in model (12), we note that the gradient of *R*_1_(**f**) is Lipschitz continuous with a constant upper bound by ||**P**||^2^||**F**||^2^. Therefore, we employ the popular forward-backward (FB) splitting algorithm [15–18] to solve model (12), then the recursive form of FB splitting becomes

$$f^{\left(k+1\right)}=prox_{\tau \;R_{2}}\left(\;f^{\left(k\right)}-\tau \;\nabla R_{1}(f^{\left(k\right)})\;\right),$$ (16)

where ∇*R*_1_(**f** ^(k)^) = **A** ^T^(**Af** ^(k)^−**g**) is the gradient of function *R*_1_ at **f** ^(k)^ and the proximal step size *τ*∈(0,2/(||**P**||^2^||**F**||^2^)). From the numerical analysis point of view, the gradient descent step within the parentheses is the forward step, and the application of proximity operator $prox_{\tau \;R_{2}}$ is the backward step.

By denoting **z** ^(k)^ = **f** ^(k)^−*τ* **A** ^T^(**Af** ^(k)^−**g**) and *α* = *τλ*, then Eq (16) becomes

$$f^{\left(k+1\right)}=\underset{f}{\text{arg min}}\frac{1}{2}||f-z^{\left(k\right)}|{|}_{2}^{2}+\alpha ||\Lambda_{2}V_{2}f|{|}_{1,2}.$$ (17)

Specially, the computation of proximity operator $prox_{\tau \;R_{2}}$ described in Eq (17) can be considered as an HDTV2-based denoising problem with **z** ^(k)^ being the noisy observation. Considering that **f** is a digital image whose intensities are finite and bounded, we can equivalently convert the unconstrained denoising problem Eq (17) to a constrained one, which is formulated as

$$\begin{array}{l} f^{\left(k+1\right)}=\underset{f}{\text{arg min}}\frac{1}{2}||f-z^{\left(k\right)}|{|}_{2}^{2}+\alpha ||\Lambda_{2}V_{2}f|{|}_{1,2}+\;\iota_{S}(f)\\ \;=\underset{f\in S}{\text{arg min}}\frac{1}{2}||f-z^{\left(k\right)}|{|}_{2}^{2}+\alpha ||\Lambda_{2}V_{2}f|{|}_{1,2}, \end{array}$$ (18)

where *S* = {**f**∈ℜ^*N*^|**f**_*i*_∈[*a*,*b*],∀*i* = 1,⋯, *N*} is a convex closed set which enforces bounded constraint on the solution, *a* and *b* are two constants, and *ι*_*s*_(**f**) is the indicator function of *S* which takes value 0 for **f**∈*S* and ∞ otherwise.

As referred in [19], || ⋅ ||_∞,2_ is the dual norm of || ⋅ ||_1,2_, then the HDTV2 regularizer in Eq (18) can be equivalently written as

$$||\Lambda_{2}V_{2}f|{|}_{1,2}=\underset{\omega \;\in B_{2}}{\text{max}}{\langle \Lambda_{2}V_{2}f,\omega \rangle }_{X_{2}}=\underset{\omega \;\in B_{2}}{\text{max}}{\langle f,V_{2}^{Τ}\Lambda_{2}^{Τ}\omega \rangle }_{2},$$ (19)

where *B*_2_ = {**ω** = (**ω**_1_,**ω**_2,⋯,_ **ω**_*N*_)∈*X*_2_| ||**ω**_*i*_ ||_2_ ≤1,∀*i* = 1,⋯, *N*} denotes the *l*_∞_−*l*_2_ unit norm ball, and 〈 ⋅,⋅ 〉_2_ denotes the inner product in Euclidean space ℜ^*N*^. Hereafter, we denote **Η**_2_ = **Λ**_2_ **V**_2_ for simplicity.

Thus, using Eq (19), we can solve the problem Eq (18) by the following minimax problem:

$$\underset{f\in S}{\text{min}}\underset{\omega \;\in B_{2}}{\text{max}}\left\{E(f,\omega )=\frac{1}{2}||f-z^{\left(k\right)}|{|}_{2}^{2}+\alpha \;{\langle f,H_{2}^{Τ}\omega \rangle }_{2}\right\}.$$ (20)

Noting that the function *E*(**f**,**ω**) can be also written as

$$E(f,\omega )\;=\frac{1}{2}||f-(z^{\left(k\right)}-\alpha \;H_{2}^{Τ}\omega )|{|}_{2}^{2}+\frac{1}{2}||z^{\left(k\right)}|{|}_{2}^{2}-\frac{1}{2}||z^{\left(k\right)}-\alpha \;H_{2}^{Τ}\omega |{|}_{2}^{2}.$$ (21)

Due to the fact that *E*(**f**,**ω**) is strictly convex in **f** and concave in **ω**, we can exchange the order of the minimum and maximum. Then, the saddle-point $\left(f^{\left(k+1\right)},\hat{\omega }\right)$ of Eq (20), which makes

$$\underset{f\in S}{\text{min}}\underset{\omega \;\in B_{2}}{\text{max}}E(f,\omega )=E(f^{\left(k+1\right)},\hat{\omega })=\underset{\omega \in B_{2}}{\text{max}}\underset{f\in S}{\text{min}}E(f,\omega ),$$ (22)

can be obtained by solving

$$f^{\left(k+1\right)}=\underset{f\;\in S}{\text{arg min}}\left\{\;\underset{\omega \;\in B_{2}}{\text{max}}E(f,\omega )\;\right\}=P_{S}(z^{\left(k\right)}-\alpha \;H_{2}^{Τ}\hat{\omega }),$$ (23)

where *P*_*S*_ denotes the orthogonal projection onto the convex closed set *S*, and $\hat{\omega }$ is the maximizer of the dual problem, which is formulated as

$$\hat{\omega }=\underset{\omega \;\in B_{2}}{\text{arg max}}\left\{\;h\;(\omega )=E\;(P_{S}(z^{\left(k\right)}-\alpha \;H_{2}^{Τ}\omega ),\omega )\;\right\}.$$ (24)

Since the objective function *h*(**ω**) in Eq (24) is differentiable and has Lipschitz continuous gradient, as detailed in [20], we can compute the gradient of *h*(**ω**) as

$$\nabla h(\omega )=\alpha \;H_{2}P_{S}(z^{\left(k\right)}-\alpha \;H_{2}^{Τ}\omega ).$$ (25)

In the following proposition, we will derive an upper bound of the Lipschitz constant of ∇*h*(**ω**).

#### Proposition 2

Let *L*(*h*) denotes the Lipschitz constant of ∇*h*(**ω**) of the dual objective function defined in Eq (24). Then, it holds that

$$L(h)\leq 24\alpha^{2}.$$ (26)

**Proof**: As referred in [21], the projection operator *P*_*S*_ onto the convex closed set *S*⊂ℜ^*N*^ is firmly non-expansive, which means that

$$||P_{S}(y_{1})-P_{S}(y_{2})|{|}_{2}\;\leq \;||y_{1}-y_{2}|{|}_{2},\;\forall y_{1},y_{2}\in {ℜ}^{N}.$$ (27)

For any pair of variables **u** and **ω**∈*X*_2_, we can obtain that

$$\begin{array}{l} ||\nabla h(u)-\nabla h(\omega )|{|}_{X_{2}}=\;||\alpha \;H_{2}P_{S}(z^{\left(k\right)}-\alpha \;H_{2}^{Τ}u)-\alpha \;H_{2}P_{S}(z^{\left(k\right)}-\alpha \;H_{2}^{Τ}\omega )|{|}_{X_{2}}\\ \;\leq \;\alpha \;||H_{2}||\;||P_{S}(z^{\left(k\right)}-\alpha \;H_{2}^{Τ}u)-P_{S}(z^{\left(k\right)}-\alpha \;H_{2}^{Τ}\omega )|{|}_{2}\\ \;\leq \;\alpha \;||H_{2}||\;||\alpha \;H_{2}^{Τ}(u-\omega )|{|}_{2}\\ \;\leq \;\alpha^{2}\;||H_{2}||\;||H_{2}^{Τ}||\;||u-\omega |{|}_{2}\\ \;=\;\alpha^{2}\;||H_{2}|{|}^{2}\;||u-\omega |{|}_{2}. \end{array}$$ (28)

Now, we compute the upper bound of ||**H**_2_||. Because $\left||H_{2}|{|}^{2}=\;||H_{2}^{Τ}H_{2}|\right|$ and **H**_2_, = **Λ**_2_ **V**_2_ we then have

$$\begin{array}{l} ||H_{2}^{Τ}H_{2}f|{|}_{2}=\;||V_{2}^{Τ}\Lambda_{2}^{Τ}\Lambda_{2}V_{2}f|{|}_{2}\\ \;=\;||(\frac{3}{8}\Delta_{xx}^{Τ}\Delta_{xx}+\frac{1}{8}\Delta_{xx}^{Τ}\Delta_{yy}+\frac{1}{2}\Delta_{xy}^{Τ}\Delta_{xy}+\frac{1}{8}\Delta_{yy}^{Τ}\Delta_{xx}+\frac{3}{8}\Delta_{yy}^{Τ}\Delta_{yy})\;f|{|}_{2}\\ \;\leq \;(\;\frac{3}{8}||\Delta_{xx}|{|}^{2}+\frac{1}{4}||\Delta_{xx}||\;||\Delta_{yy}||+\frac{1}{2}||\Delta_{xy}|{|}^{2}+\frac{3}{8}||\Delta_{yy}|{|}^{2})\;||f|{|}_{2}, \end{array}$$ (29)

where Δ_*ij*_:ℜ^*N*^→ℜ^*N*^ is the discrete second degree differential operator with respect to the subscript and its corresponding adjoint operator is given by $\Delta_{i\;j}^{Τ}:{ℜ}^{N}\to {ℜ}^{N}$ , *i*, *j*∈{*x*, *y*}.

Then, using the definitions of the second degree forward differences defined in [14], we are easy to have that each of ||Δ_*xx*_||, ||Δ_*xy*_|| and ||Δ_*yy*_|| is smaller than or equal to 4. Substituting it back to Eq (29), we have that $||H_{2}||\;\leq 2\sqrt{6}$ , and we conclude that an upper bound of the Lipschitz constant of ∇*h*(**ω**) will be *L*(*h*)≤*α* ^2^||**H**_2_||^2^ ≤ 24*α* ^2^.

To accelerate the convergence rate, we use the Nesterov’s iterative method [22] to solve problem Eq (24), which exhibits convergence rates of one order higher than the standard gradient ascent method. In detail, we employ the operator $P_{B_{2}}$ that returns the orthogonal projection onto the *l*_∞_−*l*_2_ unit norm ball *B*_2_ to compute the solution of the dual problem Eq (24), and then obtain the solution of Eq (18) by using Eq (23).

In summary, a detailed description of our complete forward-backward splitting (FBS) algorithm for HDTV2-based image reconstruction is provided in Table 1.

**Table 1:** The FBS algorithm for HDTV2-based image reconstruction.

**Input: g**, **A** = **PF**, **H**_2_ = **Λ**_2_ **V**_2_, *λ*, *τ*∈(0,2/(||**P**||^2^||**F**||^2^)), *α* = *τλ*, *Maxiter* and *Iter*. Set *k* = 1, *m* = 1 and initialize **f** ^(1)^ = **A** ^T^ **g**, **v** ^(1)^ = **ω** ^(0)^ = **0**, *t* ^(1)^ = 1.
**for** *k* = 1 **to** *Maxiter* **do**
1. forward step: **z** ^(*k*)^ = **f** ^(*k*)^−*τ* **A** ^T^(**Af** ^(*k*)^−**g**).
2. backward step: computing $f^{\left(k+1\right)}=prox_{\alpha \;||\Lambda_{2}V_{2}•|{|}_{1,2}}\left(\;z^{\left(k\right)}\;\right)$ as follows
**for** *m* = 1 **to** *Iter* **do**
$\begin{array}{l} \;\omega^{\left(m\right)}\leftarrow P_{B_{2}}\;(v^{\left(m\right)}+\frac{1}{24\alpha }H_{2}P_{S}(z^{\left(k\right)}-\alpha \;H_{2}^{Τ}v^{\left(m\right)}))\;;\\ \;t^{\left(m+1\right)}\leftarrow \frac{1+\sqrt{1+4{\left(t^{\left(m\right)}\right)}^{2}}}{2}\;;\\ \;v^{\left(m+1\right)}\leftarrow \omega^{\left(m\right)}+\frac{t^{\left(m\right)}-1}{t^{\left(m+1\right)}}(\omega^{\left(m\right)}-\omega^{\left(m-1\right)})\;; \end{array}$
and obtain $f^{\left(k+1\right)}=P_{S}(z^{\left(k\right)}-\alpha \;H_{2}^{Τ}\omega^{\left(Iter\right)}).$
Output: $\hat{f}$ - An optimal solution of Eq (12).

### Convergence Analysis

In this section, we focus on analyzing the convergence of the proposed FBS algorithm for HDTV2-based image reconstruction problem relying on the properties of averaged and firmly non-expansive operators, which is very different from the convergence proof of the FBS algorithm [17]. To simplify our analysis, according to the iteration form of FBS in Eqs (16) and (17), we first introduce the following notations:

$$\left\{ \begin{array}{l} \;S_{G}(f^{\left(k\right)})\;\overset{\Delta }{=}\;z^{\left(k\right)}\;=\;f^{\left(k\right)}-\tau \;A^{Τ}(Af^{\left(k\right)}-g)\\ S_{HDTV2}(z^{\left(k\right)})\;\overset{\Delta }{=}\;f^{\left(k+1\right)}=\underset{f\in S}{\text{arg min}}\frac{1}{2}||f-z^{\left(k\right)}|{|}_{2}^{2}+\alpha ||\Lambda_{2}V_{2}f|{|}_{1,2} \end{array}\right.,$$ (30)

for k = 1,2,…. Furthermore, we have the following relationship:

$$f^{\left(k+1\right)}=T(f^{\left(k\right)}),$$ (31)

where $T(\cdot )\;\overset{\Delta }{=}\;S_{HDTV2}(S_{G}(\cdot ))$ . Specifically, Eq (31) indicates that **f** ^(k)^ = *T* ^*k*^ **(f** ^(0)^ **)**, for any initial vector **f** ^(0)^.

It is clear from Eq (30) that *S*_*G*_(·) = (*I*−*τ*∇*R*_1_)(·) and *S*_*HDTV2*_(·) = *prox*_*αR*2_(·).

According to the well-known Krasnoselskii-Mann (KM) theorem [21], if *T* is an averaged non-expansive operator and has fixed points, then the sequence {*T* ^*k*^(**x** ^(0)^)}_*k*_ converges to a fixed point of *T*, for any initial vector **x** ^(0)^.

In order to analyze the convergence of the proposed FBS algorithm, we need to show that the operator *T* defined in Eq (31) is averaged non-expansive and the set of the fixed points of *T* is nonempty.

Based on the definitions of the averaged and firmly non-expansive operators [23], we will show that the operators *S*_*G*_, *S*_HDTV2_ and *T* are averaged non-expansive, respectively. As we know, the function *R*_1_:ℜ^*N*^→ℜ is convex and differentiable, and ∇*R*_1_ is *L*-Lipschitz continuous with *L* = ||**P**||^2^||**F**||^2^, then we can obtain that $\frac{1}{L}\nabla R_{1}$ is a non-expansive operator.

Specifically, we can rewrite that

$$S_{G}=I-\tau \;\nabla R_{1}=I-\tau \;L\;(\frac{1}{L}\nabla R_{1})=(1-\tau \;L)I+\tau \;L\;(I-\frac{1}{L}\nabla R_{1}),$$ (32)

and $I-\frac{1}{L}\nabla R_{1}$ is also non-expansive, thus *S*_*G*_ is *τL*-averaged non-expansive.

More specifically, the operator *S*_*HDTV2*_ defined in Eq (30) is the proximity operator of *αHDTV*_2_(**f**) and the proximity operators are also firmly non-expansive [15], then, we obtain that the operator *S*_*HDTV2*_ is $\frac{1}{2}$ -averaged non-expansive.

#### Lemma 1

([24]) Let *P*_1_ and *P*_2_ be *β*_1_-averaged and *β*_2_-averaged non-expansive operators, respectively, then *P*_1_ *P*_2_ is (*β*_1_+*β*_2_−*β*_1_+*β*_2_)-averaged non-expansive.

Based on Lemma 1, we can obtain that the operator *T* defined in Eq (31) is $\frac{1+\tau \;L}{2}$ -averaged non-expansive.

Next, we will show that the objective function *R*(**f**) described in Eq (12) is coercive under certain conditions and the set of the fixed points of *T* is nonempty in the following Lemma.

#### Lemma 2

Let **D**_*hh*_, **D**_*hv*_ and **D**_*vv*_ ∈ℜ^*N×N*^ be the second order difference matrices in the horizontal-horizontal direction, horizontal-vertical direction and vertical-vertical direction, respectively, and denote $D=(D_{hh};\sqrt{2}D_{hv};D_{vv})\in {ℜ}^{3N\times N}$ . Then, the objective function *R*(**f**) is coercive if *Null*(**A**)∩*Null*(**D**) = {**0**}, where *Null*(·) is the null space of the corresponding matrix and **0**∈ℜ^*N*^.

**Proof**. It is clear that **D** is not a full-rank matrix. Thus, the null space of **D** is the set {*c* **1**} with *c* being a scalar, where **1**∈ℜ^*N*^. With the HDTV2 regularizer described in Eq (11), we further have that

$$\begin{array}{l} HDTV_{2}(f)\;=\sum_{i=1}^{N}\sqrt{[3{\left(D_{hh}f\right)}_{i}^{2}+2{\left(D_{hh}f\right)}_{i}{\left(D_{vv}f\right)}_{i}+4{\left(D_{hv}f\right)}_{i}^{2}+3{\left(D_{vv}f\right)}_{i}^{2}]/8}\\ \;\geq \sum_{i=1}^{N}\sqrt{[3{\left(D_{hh}f\right)}_{i}^{2}-{\left(D_{hh}f\right)}_{i}^{2}-{\left(D_{vv}f\right)}_{i}^{2}+4{\left(D_{hv}f\right)}_{i}^{2}+3{\left(D_{vv}f\right)}_{i}^{2}]/8}\\ \;=\frac{1}{2}\sum_{i=1}^{N}\sqrt{\left[{\left(D_{hh}f\right)}_{i}^{2}+2{\left(D_{hv}f\right)}_{i}^{2}+{\left(D_{vv}f\right)}_{i}^{2}\right]}\\ \;\geq \frac{1}{2}\sum_{i=1}^{N}\frac{1}{\sqrt{3}}[\;\left|{\left(D_{hh}f\right)}_{i}\right|+\sqrt{2}\left|{\left(D_{hv}f\right)}_{i}\right|+\left|{\left(D_{vv}f\right)}_{i}\right|\;]\\ \;=\frac{1}{2\sqrt{3}}||Df|{|}_{1}\geq \frac{1}{2\sqrt{3}}||Df|{|}_{2}, \end{array}$$ (33)

where |·| denotes the absolute value of corresponding scalar and ||·||_1_ denotes the *l*_1_ norm in Euclidean space.

Using Eq (33), we obtain that

$$R(f)\geq \frac{1}{2}||g-Af|{|}_{2}^{2}+\frac{\lambda }{2\sqrt{3}}||Df|{|}_{2}.$$ (34)

Let

$$\left(\begin{array}{l} y_{1}\\ y_{2} \end{array}\right)=\left(\begin{array}{l} A\\ D \end{array}\right)\;f\overset{\Delta }{=}Uf,$$ (35)

then we can have that *Null*(**U**) = {**0**}, i.e. *Null*(**A**)∩*Null*(**D**) = {**0**}. As if for arbitrary **f′**∈*Null*(**U**), then **Af′** = **0** and **Df′** = **0**. Based on the null space of **D** is the set {*c* **1**}, **A** = **PF**∈*C* ^*M×N*^ is the sensing matrix with **F**∈*C* ^*N×N*^ denoted the two-dimensional discrete Fourier transform (DFT) matrix and **P**∈*C* ^*M×N*^ denoted the selection matrix containing *M* rows of the identity matrix of order *N*(Here we note that **P** must include the row corresponding to the (0,0) Fourier coefficient), then we have **f′** = *c* **1** but **Af′** ≠ **0** when *c* ≠ 0, thus **f′** = **0**, i.e. *Null*(**U**) = {**0**}. Then, matrix **U** is full rank. Thus, it follows that when ||**f**||_2_→∞ in Eq (34), either ||**y**_1_||_2_→∞ or ||**y**_2_||_2_→∞, then *R*(**f**)→∞. Based on that, Lemma 2 follows.

Based on Lemma 2, we obtain that the objective function *R*(**f**) is coercive and the set of the minimizers of *R*(**f**) is nonempty. Denoting $\hat{f}$ as a minimizer of *R*(**f**) and using Eq (30), we have $\hat{f}=S_{HDTV2}(S_{G}(\hat{f}))=T(\hat{f})$ so that $\hat{f}$ is a fixed point of *T*. Thus, the set of the fixed points of *T* is nonempty.

Based on above analysis, we obtain that the operator *T* is $\frac{1+\tau \;L}{2}$ -averaged non-expansive and the set of the fixed points of *T* is nonempty. According to the KM theorem [21], we can have that the sequence {**f** ^(*k*)^}_*k*_ generated by Eq (30) converges to a fixed point of *T* as *k*→∞, for any initial vector **f** ^(0)^∈ℜ^*N*^.

## Experimental Results

In this section, we present many numerical experiments to illustrate the performance of our proposed FBS algorithm for HDTV2-based image CS reconstruction problem. Specifically, we compare our proposed FBS method for HDTV2 (denoted by HDTV2-FBS) with the fast iterative shrinkage-thresholding algorithm (FISTA) for TV [18] (denoted by TV-FISTA) and state-of-the-art MM method for HDTV2 [12] (denoted by HDTV2-MM). Finally, the reconstruction performance of each method is evaluated in terms of peak signal to noise ratio (PSNR) and structural similarity index (SSIM), which are defined as

$$\text{PSNR}=10{\text{log}}_{10}\left(\frac{\underset{i,j}{\text{max}}|f(i,j){|}^{2}}{\frac{1}{rc}||f-\hat{f}|{|}_{F}^{2}}\right),$$ (36)

$$\text{SSIM}=\frac{\left(2\mu_{\hat{f}}\mu_{f}+c_{1})(2\sigma_{\hat{f}\;f}+c_{2}\right)}{\left(\mu_{\hat{f}}^{2}+\mu_{f}^{2}+c_{1})(\sigma_{\hat{f}}^{2}+\sigma_{f}^{2}+c_{2}\right)},$$ (37)

where $\hat{f}$ and *f*∈ℜ^r×c^ are the reconstructed image and the original image, respectively, ||·||_*F*_ is the Frobenius norm, $\mu_{\hat{f}}$ and *μ*_*f*_ are the average values of $\hat{f}$ and *f*, respectively, $\sigma_{\hat{f}}^{2}$ and $\sigma_{f}^{2}$ are the variance of $\hat{f}$ and *f*, respectively, $\sigma_{\hat{f}\;f}$ is the covariance of $\hat{f}$ and *f*, *c*_1_ and *c*_2_ are two small positive constants.

### Experimental Setting

In our experiments, the test images including magnetic resonance (MR) image and cell images are Brain, Fluorescent cell, CIL 240 and CIL 248, respectively, where the Brain image is available at http://www.strokeeducation.co.uk/?page_id=300, the Fluorescent cell image is available at http://rsb.info.nih.gov/ij, and the CIL 240 and CIL 248 images are part of the biomedical image database [25]. Moreover, the intensities of all test images are normalized in the range of [0,1]. Specifically, the compressive samples for each test are acquired by first applying variable density random Fourier encoding and then adding the Gaussian white noise corresponding to a SNR level of 30 dB.

To compare the performance fairly, we constrain the reconstructed images of all methods to lie in the convex closed set *S* = {**f**∈ℜ^*N*^ |**f**_i_∈[0,1],∀*i* = 1,…, *N*}. Moreover, all parameters of the TV-FISTA and HDTV2-MM methods are set to be the default values in [18], [12], but the regularization parameter for each method is chosen to give the best PSNR results. All the methods are implemented in MATLAB 7.11.0 (R2010b) and run on an Intel (R) Xeon(R) CPU 2.67-GHz PC with 4-GB memory.

### Result Analysis

To characterize the performance quantitatively, we provide the PSNR, SSIM and running time results of different methods under different sampling ratios in Table 2. It is clear to observe that the proposed HDTV2-FBS method always provides the best PSNR and SSIM results, where the PSNR result of HDTV2-FBS method is even 1 dB higher than the HDTV2-MM method for the case of the CIL 240 image. The TV-FISTA method provides very competitive PSNR and SSIM results with the HDTV2-MM method, while the TV-FISTA method takes much less running time than the HDTV2-MM method. Considering the comparisons of running time, the proposed HDTV2-FBS method always takes much less running time than the HDTV2-MM method. More specifically, the TV-FISTA method consistently takes the shortest running time. In fact, the computation of the HDTV2-FBS method is more complex than that of the TV-FISTA method, thus it is more valuable for our proposed HDTV2-FBS method to spend a little more time than the TV-FISTA method for achieving better reconstruction results. Based on these numerical results, they clearly demonstrate that our proposed HDTV2-FBS method is more effective and efficient for image CS reconstruction.

**Table 2:** PSNR (dB), SSIM and running time (s) results of different methods on test images.

Sampling Ratio (%)	20			30			50		
	PSNR	SSIM	Time	PSNR	SSIM	Time	PSNR	SSIM	Time
	**Brain (256×256)**								
TV-FISTA	33.51	0.9039	10.71	35.15	0.9226	10.60	37.97	0.9535	9.21
HDTV2-MM	33.07	0.8921	67.62	35.07	0.9224	77.05	38.08	0.9551	64.32
HDTV2-FBS	**33.91**	**0.9095**	20.90	**35.66**	**0.9313**	19.87	**38.47**	**0.9576**	18.21
	**Fluorescent cell (225×225)**								
TV-FISTA	30.95	0.8538	9.12	32.88	0.8952	7.87	36.67	0.9524	7.16
HDTV2-MM	30.75	0.8533	49.71	32.88	0.8982	50.84	36.73	0.9534	48.90
HDTV2-FBS	**30.96**	**0.8589**	16.00	**32.96**	**0.9003**	15.07	**36.90**	**0.9548**	14.38
	**CIL 240 (256×256)**								
TV-FISTA	22.16	0.7405	10.66	24.08	0.8275	10.74	28.48	0.9206	10.28
HDTV2-MM	21.48	0.6664	48.64	23.32	0.7502	53.58	27.49	0.8723	55.78
HDTV2-FBS	**22.40**	**0.7606**	23.61	**24.32**	**0.8424**	21.79	**28.74**	**0.9275**	20.40
	**CIL 248 (256×256)**								
TV-FISTA	33.66	0.9130	10.29	35.80	0.9403	9.52	38.95	0.9658	8.92
HDTV2-MM	33.22	0.9011	71.38	35.46	0.9324	63.30	38.75	0.9606	55.44
HDTV2-FBS	**33.79**	**0.9155**	24.29	**36.05**	**0.9431**	21.00	**39.31**	**0.9684**	18.80

Next, from both reconstruction quality and convergence speed point of view, we representatively show some examples with different sampling ratios to illustrate the performance of our proposed HDTV2-FBS method.

**Fig 1:**
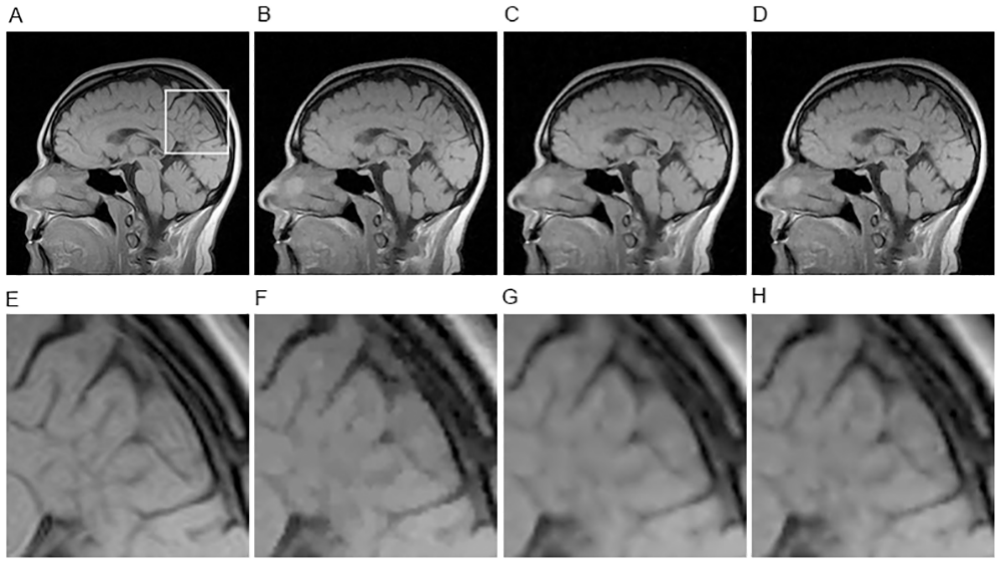
The reconstructed results of different methods on Brain image from noisy and undersampled Fourier data (noise level: 30 dB and sampling ratio: 20%). (A) Original image; (B) TV-FISTA result; (C) HDTV2-MM result; (D) HDTV2-FBS result; (E) the zoomed version of region marked by the white box in original image; (F) the zoomed version of TV-FISTA result; (G) the zoomed version of HDTV2-MM result; (H) the zoomed version of HDTV2-FBS result.

**Fig 2:**
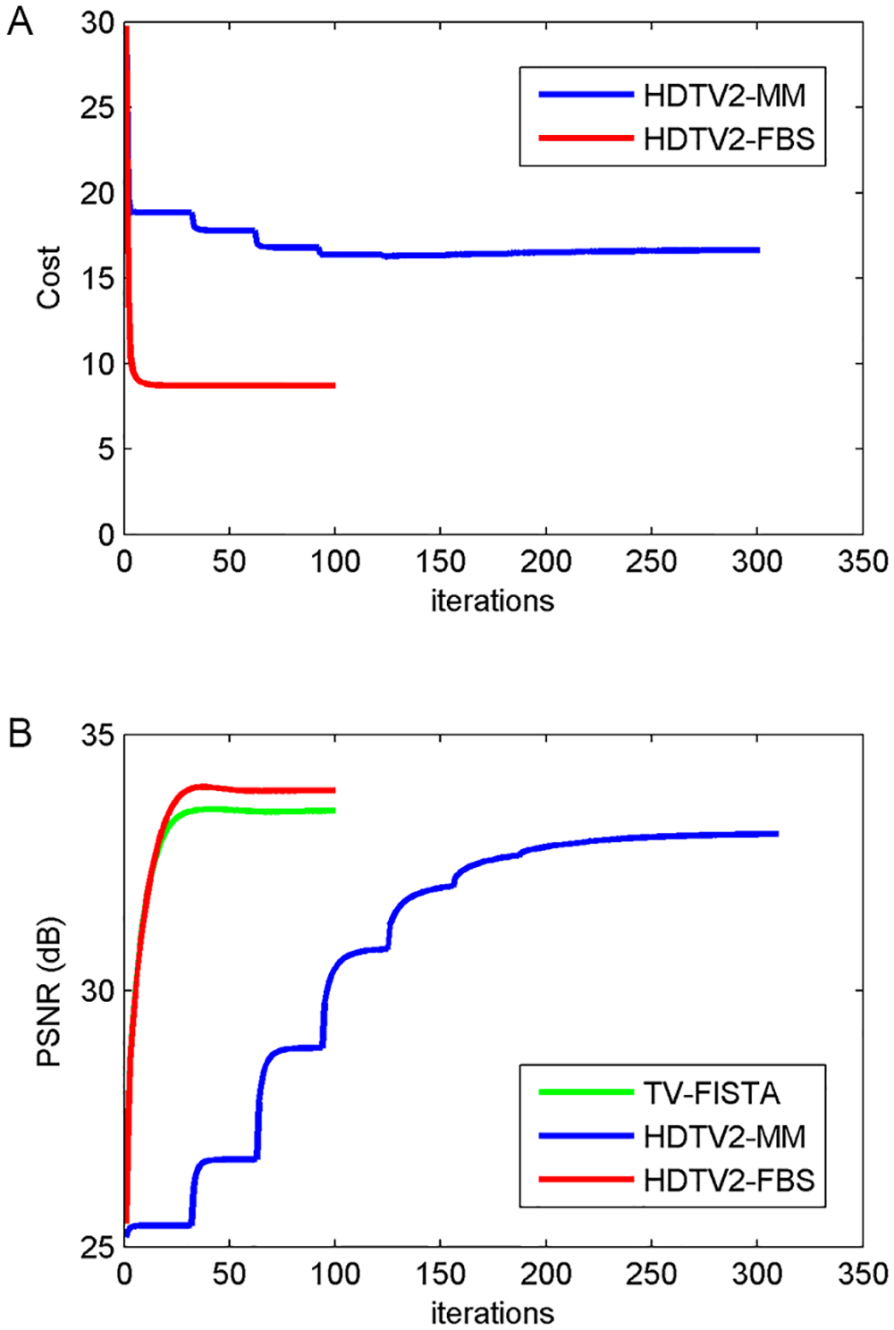
The convergence curve of the cost of objective function and PSNR results versus iterations corresponding to the reconstructed example shown in Fig 1. (A) Cost versus iterations; (B) PSNR versus iterations.

Fig 1 shows the reconstructed images of different methods on Brain image with sampling ratio 20% and noise level 30 dB. Obviously, we observe that the HDTV2-MM method and our proposed HDTV2-FBS method can both preserve some fine details in the reconstructed images. Furthermore, in order to highlight the differences among these methods, we zoom in the corresponding region marked by the white box in Fig 1A and present the zoomed details in Fig 1E–1H. By inspecting these images carefully, the HDTV2-MM method provides better preservation of smooth image regions but loses some fine details, and our proposed HDTV2-FBS method can better preserve some fine details, while the TV-FISTA method suffers from severe blocky artifacts in smooth regions. More specifically, we plot the cost of the objective function versus the number of iterations for the HDTV2-MM and HDTV2-FBS methods in Fig 2A to illustrate which one is better for cost minimization. As shown in Fig 2A, we can obtain that the proposed HDTV2-FBS method is more accurate than the HDTV2-MM method in terms of cost minimization, where the proposed HDTV2-FBS method exhibits a much faster convergence speed than the HDTV2-MM method. Meanwhile, we also plot the convergence curve of PSNR versus the number of iterations in Fig 2B. It is clear that our proposed HDTV2-FBS method not only provides better PSNR results than the TV-FISTA and HDTV2-MM methods, but also exhibits a much faster convergence speed than the HDTV2-MM method.

**Fig 3:**
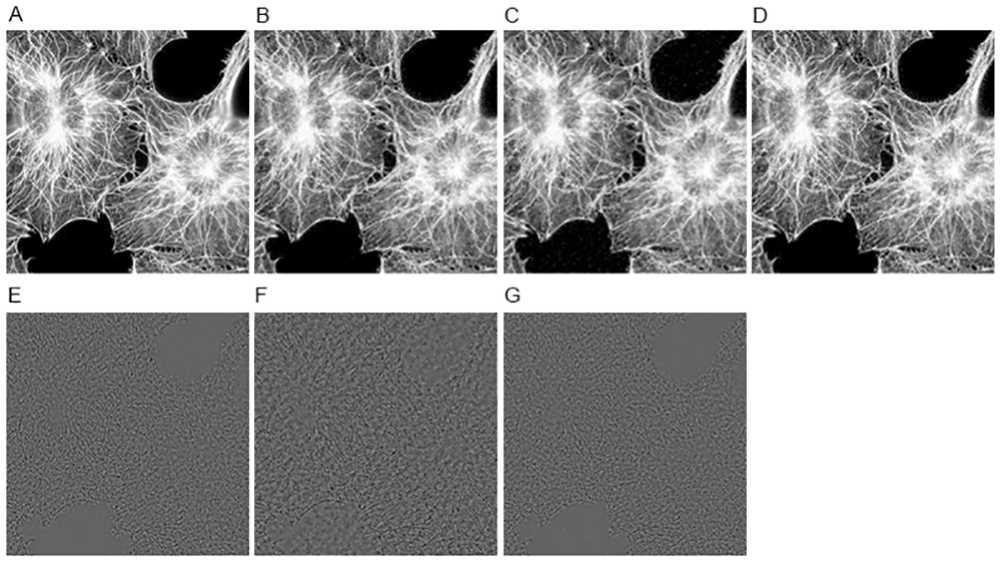
The reconstructed results of different methods on CIL 240 image from noisy and undersampled Fourier data (noise level: 30 dB and sampling ratio: 30%). (A) Original image; (B) TV-FISTA result; (C) HDTV2-MM result; (D) HDTV2-FBS result; (E) the residual of TV-FISTA result; (F) the residual of HDTV2-MM result; (G) the residual of HDTV2-FBS result.

**Fig 4:**
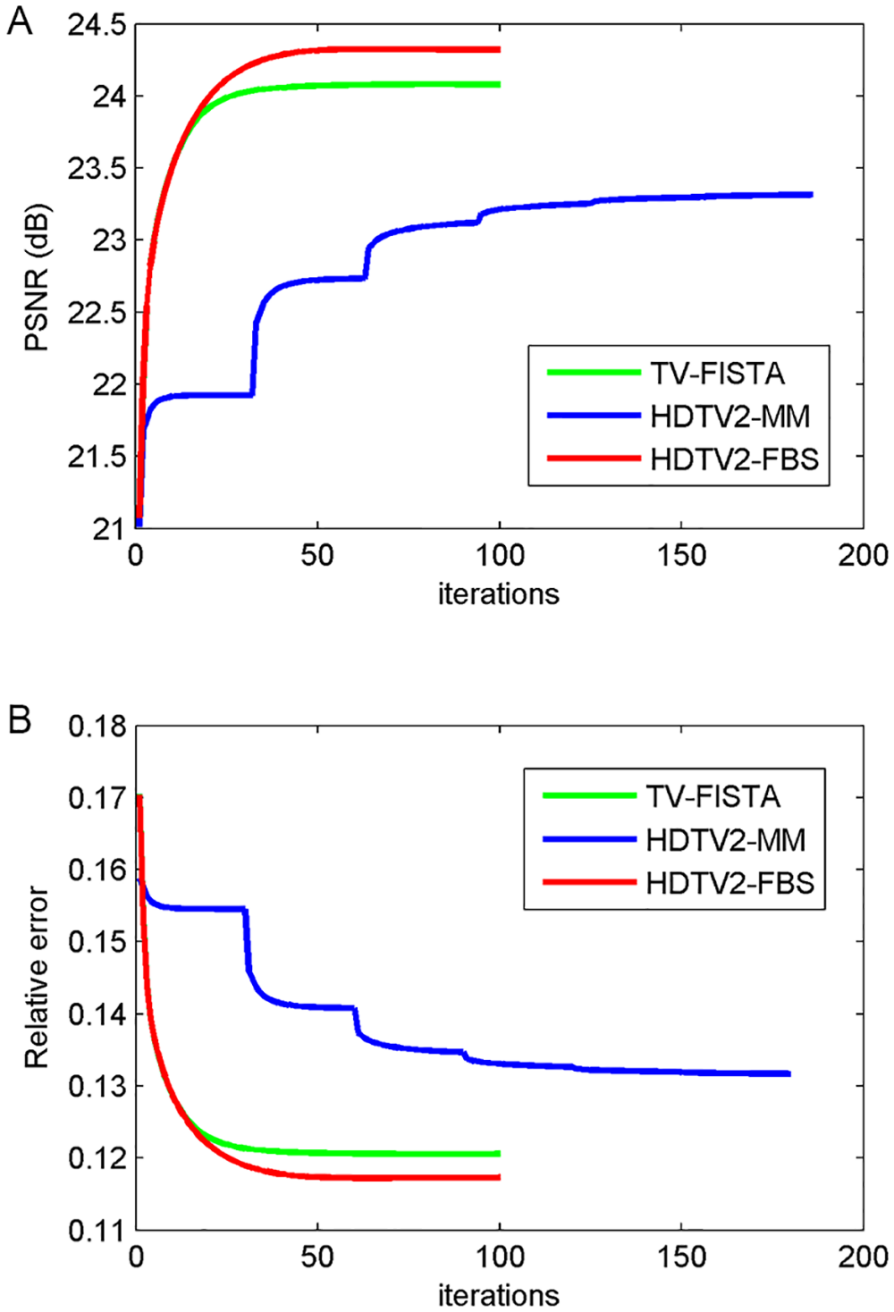
The convergence curve of PSNR and relative error results versus iterations corresponding to the reconstructed example shown in Fig 3. (A) PSNR versus iterations; (B) Relative error versus iterations.

The reconstructed results of different methods on image CIL 240 with sampling 30% and noise level 30 dB are shown in Fig 3. Since the CIL 240 image consists of many filament-like features, it is more desirable to well preserve these elongated features as far as possible. As we can clearly see from the residual images of TV-FISTA, HDTV2-MM and HDTV2-FBS results shown in Fig 3E–3G, the HDTV2-MM method could not well preserve the filament-like features and still leaves some noise in the smooth regions of the reconstructed image. The TV-FISTA method can remove the residual noise but fails to preserve filament-like features and produces some staircase effect in the smooth regions. By contrast, our proposed HDTV2-FBS method can better remove the residual noise and at the same time preserve the elongated filament-like features more sharpness, which makes the reconstructed image more natural. Meanwhile, considering the convergence curves of PSNR and relative error plotted in Fig 4, we can observe that our proposed HDTV2-FBS method provides the best PSNR and relative error results. Specifically, the proposed HDTV2-FBS method shows the fastest convergence speed, where it is about two folds faster than that of the HDTV2-MM method.

**Fig 5:**
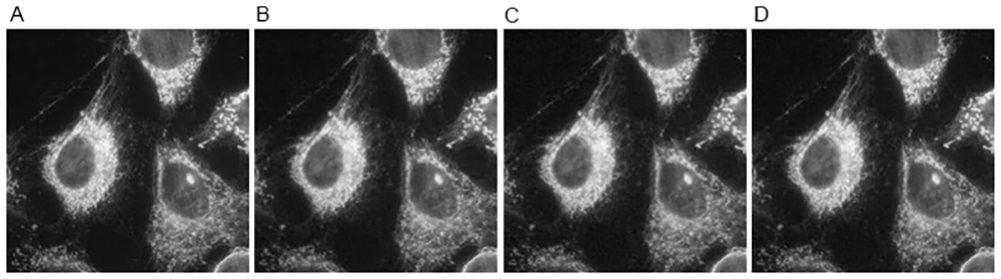
The reconstructed results of different methods on CIL 248 image from noisy and undersampled Fourier data (noise level: 30 dB and sampling ratio: 50%). (A) Original image; (B) TV-FISTA result; (C) HDTV2-MM result; (D) HDTV2-FBS result.

**Fig 6:**
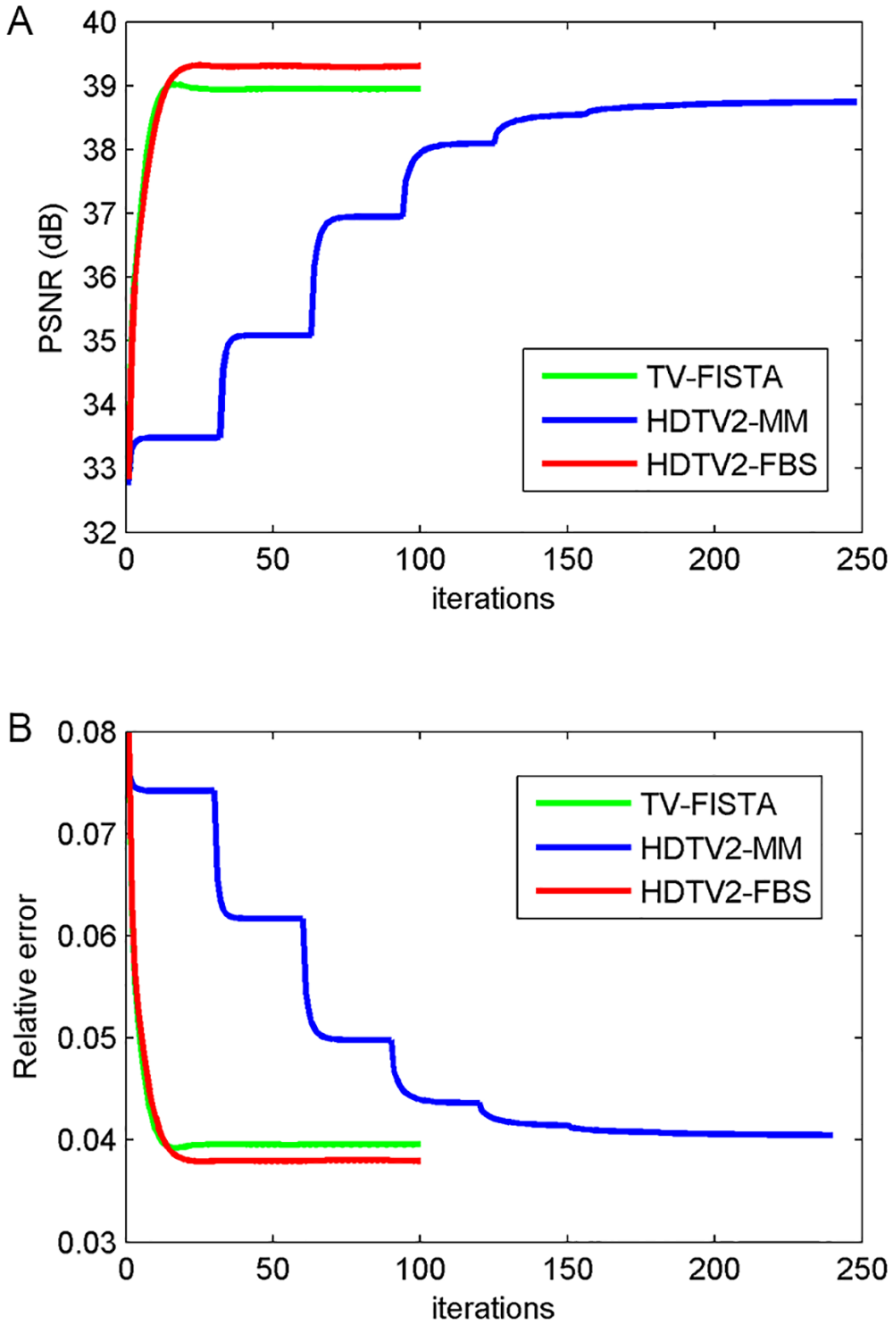
The convergence curve of PSNR and relative error results versus iterations corresponding to the reconstructed example shown in Fig 5. (A) PSNR versus iterations; (B) Relative error versus iterations.

Finally, we show the reconstructed results of different methods on image CIL 248 with sampling 50% and noise level 30 dB in Fig 5. In this sense, the sampling ratio is enough large to ensure a good reconstruction quality. We can see from the reconstructed images that the TV-FISTA method, the HDTV2-MM method and our proposed HDTV2-FBS method provide very good reconstruction of image features. Moreover, from the convergence speed and accuracy point of view, we clearly see from Fig 6 that our proposed HDTV2-FBS method provides the best PSNR and relative error results, and shows the fastest convergence speed, where the convergence speed of our proposed HDTV2-FBS method is much faster than that of the HDTV2-MM method. Thus, we can conclude that our proposed HDTV2-FBS method is more efficient for image CS reconstruction tasks in terms of accuracy and convergence speed.

## Conclusions

In this paper, we have introduced an efficient HDTV2 algorithm for image CS reconstruction using forward-backward splitting scheme. Under the spectral decomposition framework, HDTV2 is reinterpreted as a novel weighted *L*_1_-*L*_2_ mixed norm of the second degree image derivatives. Based on the resulting equivalent formulation of HDTV2, a computationally efficient forward-backward splitting scheme is thus designed to solve the HDTV2-based reconstruction problem. More specially, we prove in detail the convergence of the proposed FBS algorithm. Furthermore, many experimental results on MR image and cell images show that the proposed scheme provides better reconstruction results and especially exhibits much faster convergence speed than the iteratively reweighted MM method. Specifically, the proposed scheme can also preserve the elongated filament-like features more sharpness for cell images.
